# Aquatain® causes anti-oviposition, egg retention and oocyte melanization and triggers female death in *Aedes aegypti*

**DOI:** 10.1186/s13071-022-05202-0

**Published:** 2022-03-22

**Authors:** Hamady Dieng, Storm McLean, Holly Stradling, Cole Morgan, Malik Gordon, Whitney Ebanks, Zoila Ebanks, Alan Wheeler

**Affiliations:** 1 Mosquito Research and Control Unit (MRCU), George Town, Cayman Islands; 2 The University College of the Cayman Islands, Olympic Way, George Town, Cayman Islands; 3 The University of Liverpool, Liverpool, L69 3BX UK; 4 The Forensic Department, Health Services Authority, George Town, Cayman Islands

**Keywords:** *Aedes aegypti*, Aquatain®, Oviposition, Egg retention, Melanization, Female death

## Abstract

**Background:**

In arboviral disease systems where the virus can be transmitted from male to female vectors and from one generation to the next, targeting the female (especially when she is gravid) can help alter the persistence of the virus in nature and its transmission. A typical example is *Aedes* *aegypti*, which has become unmanageable due to the development of insecticide resistance. Despite evidence that monomolecular surface films prevent the selection of genetic resistance, their potential in *Aedes* vector control remains largely unexplored.

**Methods:**

We examined the oviposition, egg retention, oocyte melanization, and female mortality of the Cayman Islands strain of *Ae. aegypti*, using choice (balanced and unbalanced) and no-choice bioassays involving Aquatain® Mosquito Formulation (AMF; Aquatain Products Pty Ltd.), a polydimethylsiloxane–based liquid used for mosquito control.

**Results:**

When presented with similar opportunities to oviposit in two sites treated with AMF and two other sites with untreated water (control), egg deposition rates were significantly higher in the untreated water sites than in the AMF-treated sites (*P* < 0.05). We also observed a matching pattern of egg deposition preference in environments with more options in terms of AMF-treated sites. Females laid significantly more eggs when water was the only available medium than when all sites were treated with AMF (*P* < 0.05). Also, significantly more mature eggs were withheld in the AMF no-choice environment than in the no-choice test involving only water (*P* < 0.05). Internal oocyte melanization was not observed in females from the oviposition arenas with the lowest AMF presence (equal-choice and water-based no-choice); in contrast, this physiological response intensified as the number of AMF-treated sites increased. Female death occurred at high rates in AMF-treated environments, and this response increased with the increasing presence of such egg deposition sites.

**Conclusions:**

This study demonstrated that AMF acted as a deterrent signal to ovipositing *Ae. aegypti* and as an indirect adulticide. These results suggest that AMF may be a promising control tool against the dengue vector, and this warrants further evaluation under field settings.

**Graphical Abstract:**

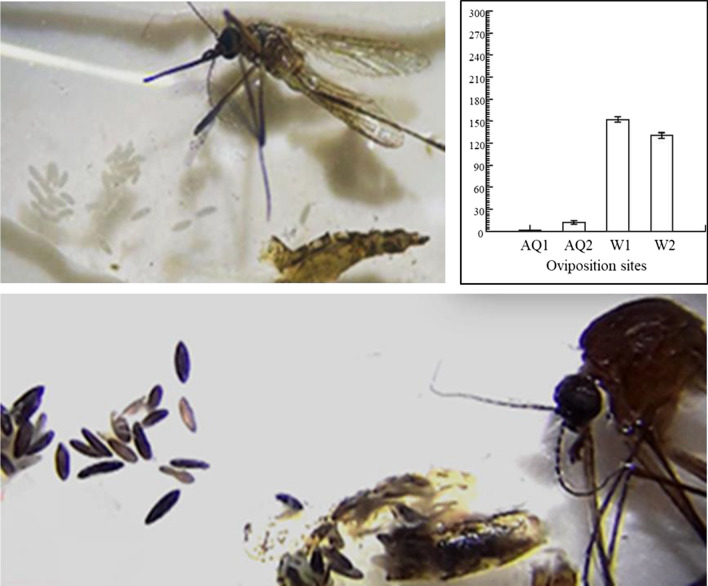

## Background

Despite several decades of control efforts, mosquito vectors and the diseases they transmit still represent significant threats to human health [1, 2]. Beyond disease transmission, mosquitoes represent a serious menace to human quality of life [3] and economic development [4]. This is the case for most Caribbean islands [5], as demonstrated by an unprecedented epidemic of chikungunya and Zika viruses and febrile illness due to dengue in recent years [6]. These public health threats, in particular, the link between the Zika virus and reproductive health, pregnancy and congenital disabilities [7–9], have stimulated interest in *Aedes aegypti*, the primary vector of all the above-mentioned viruses [10].

Efforts to prevent outbreaks of these diseases have mostly counted on the use of insecticides, but success has been limited due to the development of insecticide resistance [11, 12]. Apart from increased resistance to the four existing families of adulticides, namely pyrethroids, organophosphates, carbamates and organochlorines [13, 14], the control of *Ae. aegypti* with larvicides is hindered by a reduced susceptibility [15], international scrutiny and deregulation due to toxicity against biodiversity [16] and the existence of inconspicuous larval habitats [17, 18].

Female *Ae. aegypti* disperse eggs across multiple natural and artificial containers associated with human activity [19–21]. Despite this multitude of potential breeding sites, *Ae. aegypti* has been reported to successfully locate those that can guarantee the success of the offspring and to ignore others that are unsuitable for larval development completion [22–24]. This ability to identify high-quality oviposition sites and select those that provide increased larval performance is mediated by many parameters, but most studies have focused on the role of olfactory cues [25] and habitat physics [20]. However, while some studies have specifically analyzed components of the site's physical structure that affect egg deposition choice [26], there has been little research on the physical features of water bodies, which are known to present several visual signals [20], such as texture and reflectance from the water surface; these cues have been reported to influence the oviposition of container-breeding mosquitoes, including *Ae. aegypti* [27–30].

Surface tension is a property of water that plays an integral part in the development and survival of aquatic insects, including mosquitoes [31, 32]. Surface tension can be generated when a water surface is covered with an oil film [33, 34]; in this situation, the oil film elicits an elastic behavior in the water, causing it to acquire as small a surface area as possible [35]. This reduction in surface tension produces a film at the air–water interface [20]. Adult *Aedes aegypti* prefer a high level of surface tension and are incapable of resting on a water surface with a reduced tension [36]; specifically, it exhibits high mortality responses by drowning when exposed to water with reduced surface tension [36]**.**

In addressing dengue vector control, the World Health Organization [37] and many other scientists studying mosquitoes [23, 38] have called for oviposition to be taken into account, not only because of transovarial transmission of viruses [39] but also because preventing oviposition can disrupt the life-cycle of the mosquito and thereby reduce population growth [23]. Due to these potentially positive control outcomes, some studies have addressed the use of oil to prevent mosquito oviposition, but most of these investigations have used either essential oils [40–42], lecithin monolayers [43–45] or Agnique MMF, a monomolecular surface film formulation (Cognis Corp., Cincinnati, OH, USA) [46], all of which are known to be easily broken down by wind and rainfall [47, 48]. In a recent study [49] that has tested an oil film with high resilience to being broken down by wind and rain, namely Aquatain® Mosquito Formulation (AMR; Aquatain Products Pty Ltd, Dandenong South, VIC, Australia [50]), the authors did not specifically address oviposition and *Ae. aegypt*i. In the present study, we assessed the oviposition, egg retention, oocyte melanization, and female mortality of *Ae. aegypti* in response to the presence of different levels of AMF in potential breeding sites.

## Methods

### Colony maintenance

The *Ae. aegypti* mosquitoes used in this study were from a colony maintained at the insectarium of the Mosquito Research and Control Unit (MRCU; George Town, Cayman Islands) and maintained at 26.5 ± 1 °C, 65 ± 4% relative humidity, and a photoperiod of 13:10 h (light:dark) with 1 h of dusk. The colony originated from larvae collected from different containers in George Town in November 2020. The larvae were standardly reared in plastic trays with 800 ml tap water containing 100– 150 larvae. Larval food (ground Tetramin; Tetra, Melle Germany) was supplied once daily, and the rearing medium was replaced with fresh water prior to each third feeding [24]. Pupae were placed in polystyrene cups (50-ml capacity) and transferred to BugDorm cages (30 × 30 × 30 cm; MegaView Science Co., Ltd., Taichung, ROC [Taiwan]). Emerging adults had continuous access to 10% sucrose solution. Females were artificially offered blood meals once every 2 weeks. Three days after blood-feeding, eggs were collected, air-dried under insectary conditions and stored for colony maintenance or experiments.

### Experimental specimens

To obtain gravid females, egg samples from the colony stock were submerged in tap water, and four larval population replicates, each with 200 newly hatched larvae, were reared as outlined in section Colony maintenance. The gravid females were fed every 2 days with Tetramin, and the rearing water of each colony was replaced with fresh water before pupation started. Pupae were held in plastic cups and transferred into cages where a 10% sugar solution was available. Three- to four-day-old females were offered blood meals for 10 min. Fully engorged females with digested blood meals for 3 days were considered to be gravid and used as experimental material.

### Chemical and test concentration

The AMF used in this study contains 78% active ingredient polydimethylsiloxane (also called silicone) and was applied at 1 ml/m^2^, which is the application rate recommended by the manufacturer [50]. The test oviposition medium was generated with reference to the approved application rate and taking into account the dimensions of the experimental containers used in this study. Each container used as an oviposition site (depth: 7.3 cm, diameter: 3.3 cm) had a surface area of 33.16 cm^2^. Based on these dimensions, a volume of 3.316 µl of AMF was added to 150 ml of water, and the resulting solution was used as an oviposition medium. The control water medium had a final volume of 150 ml + 3.316 µl. For convenience, containers with the test medium or water were designated as “AQ” and “W,” respectively.

### Experimental features

**Fig. 1:**
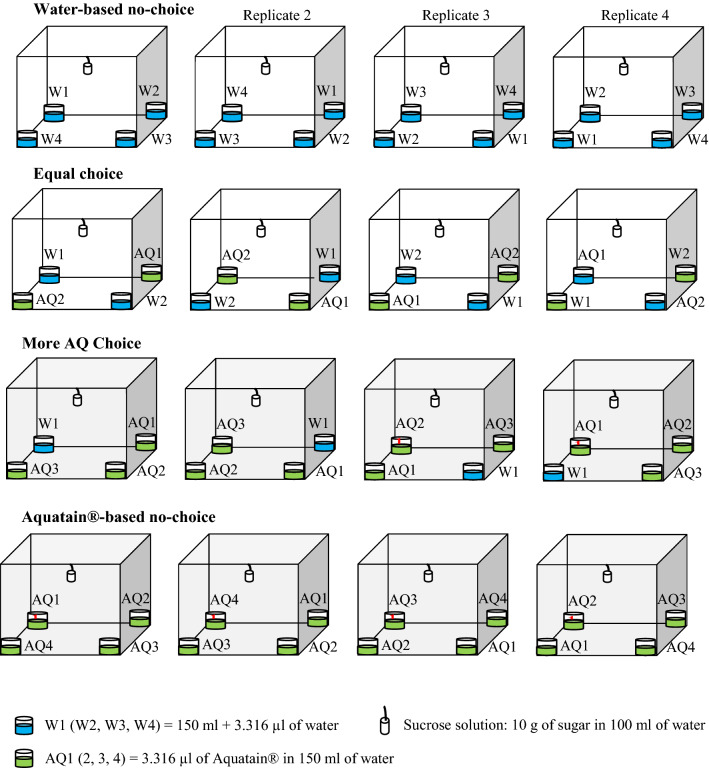
Oviposition bioassay design. The containers were placed on the dish, which was positioned at the bottom center of the cage. Containers were placed such that each container was at an identical distance from the adjacent container. A bioassay replicate coincided with one arrangement of the four containers

All oviposition tests were performed following a published experimental design [24, 51], with slight modifications. Figure 1 shows the design of the oviposition bioassay. The egg deposition space consisted of four acrylic containers (depth: 7.3 cm, diameter: 3.3 cm), each with a layer of white paper (length: 8 cm, width: 8 cm) that covered the entire surface of the container and served as an egg deposition substrate. A container was placed at each corner of the cage; to remove/minimize any likely location bias, the position of the oviposition site (i.e. the corner at which a specific container was placed) was modified in the bioassay replicates in accordance with a previously described clockwise replication blueprint [24, 51]. In this scheme, each oviposition test replicate had a designated layout regarding the four oviposition containers within the cage. All bioassay types were repeated four times, and for each replication, a batch of 12 fully blood-fed females that had digested their meals for 3 days, new egg deposition substrates and new oviposition media were used. All oviposition arenas were equipped with a sugar source supplied by a cotton wick soaked with a 10% sucrose solution. The sugar supply apparatus (cotton wick and fresh sugar solution) was replenished once during the oviposition period, which lasted 10 days. All oviposition bioassays were performed at 28 ± 2 °C and 70 ± 3% relative humidity, respectively.

### Bioassays

To ascertain whether or not the presence of AMF influences the oviposition responses of *Ae. aegypti*, we ran four consecutive bioassays (Fig. 1 [51]. In the first test, 12 gravid females were placed in a BugDorm cage (30 × 30 × 30 cm) containing an oviposition arena with four W containers (W1, W2, W3, and W4) and a sugar supply source. A second bioassay was performed, as illustrated in Fig. 1, but the 12 females were given options to lay eggs in four AQ containers (AQ1, AQ2, AQ3, and AQ4). In the third test, 12 females were given equal chances to lay eggs in two AMF-treated containers (AQ1 and AQ2) and two others with water (W1 and W2). In the fourth bioassay, 12 females were presented with more options to oviposit in AMF-treated containers (AQ1, AQ2, and AQ4) than in water (W1). Each of the tests was replicated four times, and in all bioassays, females were allowed to oviposit for 10 days.

### Data collection and statistical analyses

At the end of the oviposition period, dead females were counted in each cage replicate of each bioassay type and immediately frozen alongside survivors. Egg depositions were checked in all bioassays following the procedures of Dieng et al. [51], using a stereomicroscope (Motic SMZ-171-TLED; Motic Instruments Inc.; Schertz, TX, USA). The total number of eggs laid in each container replicate was quantified by counting the eggs present on the surface of the water media, paper substrates and lower surfaces of the bottom of the containers. The resulting numbers were used to determine the percentages of eggs laid in a given oviposition container replicate using the following formula: (total number of eggs deposited in a given media divided by the total number of eggs deposited in all media of a given bioassay replicate) × 100. The mean values of numbers of eggs deposited in a given bioassay replicate were determined by summing up all numbers of eggs in a given bioassay divided by the total number of experimental females. Percentages and mean values were utilized to score oviposition responses. Non-dried females were dissected, and the two ovaries were examined for egg presence under the stereomicroscope. The number of eggs withheld was counted for each dissected female, and the number of eggs produced by a female was defined as the sum of eggs laid and retained, in accordance with Farjana and Tuno [52]. Following Dieng et al. [51], we calculated the egg retention rate for each dissected female as: (total number of eggs withheld divided by the total of eggs produced) × 100. The percentage of eggs retained by a given female was defined as: (total number of eggs retained divided by the total number of eggs produced) × 100. At each dissection, the numbers of eggs with different coloration (white/light gray; dark-gray with black spots; and black) were recorded. We considered eggs with white/light-gray, dark-grey and black coloration eggs to be non-melanized, melanizing and melanized, respectively. The number of eggs in each state was used to determine the percentages of non-melanized, melanizing and melanized eggs. The differences in oviposition, egg retention and eggshell melanization responses were analyzed by nonparametric test (Kruskal–Wallis). The Dwass-Steel-Chritchlow-Fligner test was used to assess the significance of the differences between mean values for oviposition, egg retention and eggshell melanization. Female mortality data were checked for normality using the Shapiro–Wilk test. Mortality responses were examined by analysis of variance (ANOVA) from Systat version 13 [53], and mean mortality values were separated by the Tukey–Kramer honestly significant difference (HSD) test. In all analyses, *P* < 0.05 indicated statistical significance.

## Results

### Oviposition responses to AMF-treated water at various competition levels with water

**Fig. 2:**
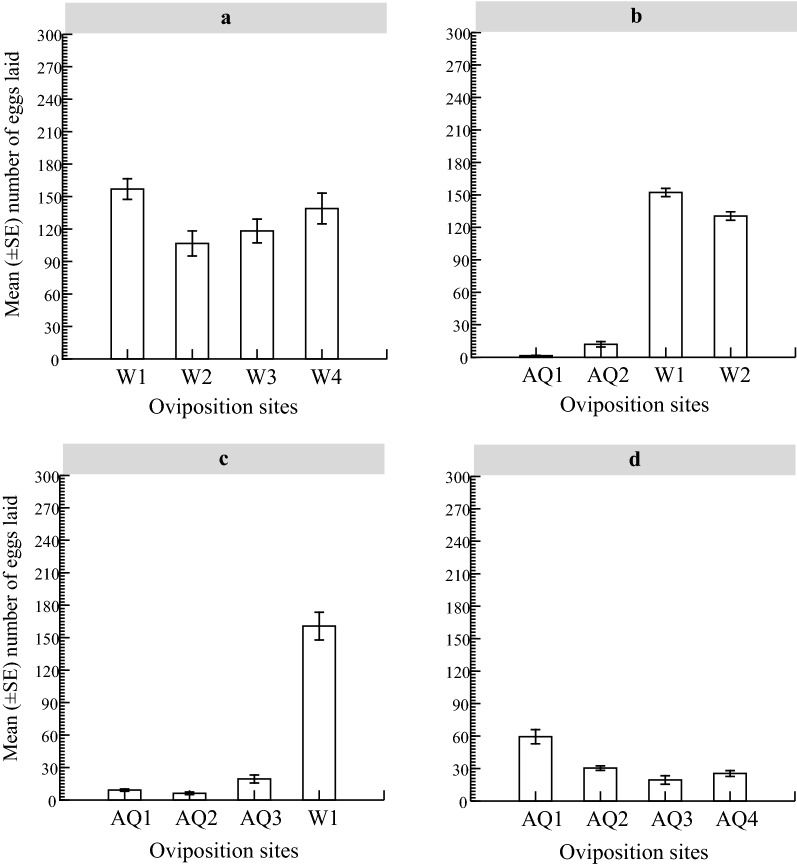
Responses of gravid *Aedes aegypti* females when given a choice to oviposit in four cups: **a** water-based no-choice, **b** equal choice, **c** more oviposition opportunities in containers with AMF-treated water, **d** AMF-based no-choice.* Abbreviations*: AMF, Aquatain® Mosquito Formulation; AQ, containers with the test medium (AMF); W, containers with water only

When water was the only medium available to the female mosquitoes, eggs were found in each of the four containers. Of the 2084 eggs laid, 30.13% (628/2084), 20.49% (427/2084), 22.69% (473/2084) and 26.68% (556/2084) were deposited in W1, W2, W3 and W4, correspondingly. The mean (± standard error [SE]) number of eggs deposited was 130.25 ± 21.31 per container (W1: 157.00 ± 37.68, range: 72–230; W2: 106.75 ± 45.87, range: 24–238; W3: 118.25 ± 43.59, range: 54–247; W4: 139.00 ± 56.25, range: 40–286). Egg deposition did not differ between these containers (Kruskal–Wallis test statistic: 0.746, *df* = 3, *P* = 0.862) (Fig. 2a). When given equal chances to oviposit in two containers holding water supplemented with AMF and two others with water, *Ae. aegypti* females laid eggs in all containers, but oviposition responses varied significantly with container medium. Of the 1185 eggs oviposited by the 12 females, 95.44% (1131/1185) were deposited in containers with water, and 4.56% (54/1185) deposited in containers with AMF-treated water. The mean egg deposition was significantly higher in containers with water (141.37 ± 10.84 eggs, range: 93–184; W1: 152.25 ± 15.19; W2: 130.50 ± 15.47) than in containers supplemented with AMF (6.75 ± 4.97 eggs, range 0–41; AQ1: 2.50 ± 0.95, range: 0–4; AQ2: 12.00 ± 9.80, range: 0–41) (Kruskal–Wallis test statistic: 11.463, *df* = 1, *P* = 0.001) (Fig. 2b). When there were more choices to lay eggs in containers with water treated with AMF, the 48 females laid 783 eggs in total, including 643 in the container with water and 140 in the three cups containing AMF, corresponding to 82.12% and 17.88% of the total eggs laid, respectively. Egg deposition was significantly lower in the presence of the oil (11.66 ± 5.13 eggs, range: 0–63; AQ1: 9.25 ± 4.38; AQ2: 6.25 ± 4.62; AQ3: 19.50 ± 14.72) than when water was the only oviposition medium (160.75 ± 50.77 eggs, range: 78–287) (Kruskal–Wallis test statistic: 8.533, *df* = 1, *P* = 0.003) (Fig. 2c). When AMF-treated containers were the only oviposition sites, *Ae.* *aegypti* deposited eggs in all four containers. A total of 540 eggs were deposited by the 48 females, of which 44.07% (238/540), 22.6% (122/540), 14.44% (78/540) and 18.88% (102/540) were oviposited in containers AQ1, AQ2, AQ3, and AQ4, respectively. Egg depositions ranged from 0 to 112 and averaged 33.75 ± 8.39 eggs per container (AQ1: 59.50 ± 25.97; AQ2: 30.50 ± 8.13; AQ3: 19.50 ± 15.65; AQ4: 25.50 ± 10.44). There were no appreciable discrepancies in oviposition response between the four containers (Kruskal–Wallis test statistic: 2.976, *df* = 3, *P* = 0.375) (Fig. 2d).

### Comparative oviposition responses relative to option availability and egg retention

In the water-based no-choice test, the 48 female mosquitoes (12 females per cage replicate) produced a total of 2734 eggs; the mean number of eggs laid individually was 43.43 (2084/48). The mean number of eggs laid individually was 24.68 (1185/48), 16.31 (783/48) and 11.25 (540/48) for *Ae. aegypti* females provided with (i) matching oviposition options and (ii) more oviposition opportunities in containers supplemented with AMF, and (iii) in the AMF-based no-choice setting, respectively. Total egg deposition when water was the only oviposition medium was 1.75-, 2.66- and 3.86-fold higher than that when half, three-fourths (3 of 4) and all (4 of 4) oviposition sites were treated with AMF, respectively (Table 1). Eggs were retained in all experimental oviposition designs (Table 2), but egg retention responses varied significantly with oviposition arena layout (Kruskal–Wallis test statistic: 23.416, *df* = 3, *P* < 0.0001). In the no-choice test involving only water, a total of 649 eggs were recovered from the ovaries of the 48 female mosquitoes at 10 days after uptake of the blood meal, indicating that these females retained 23.73% (649/2734) of their total egg production. In the equal-choice test, a total of 462 eggs were collected following dissection of the 28 surviving females at 1 week after blood digestion. When more containers were treated with the oil, a total of 1669 eggs were retained by the 41 surviving females; with 986 eggs for 38 females when containers with the oil were the only egg deposition sites. The mean number of eggs retained per female in the water-based no-choice trial (13.52 ± 3.49 eggs; range: 0–76 eggs) was appreciably lower than that in the equal-choice experiment (17.11 ± 3.99 eggs; range: 0–63 eggs) (Dwass-Steel-Chritchlow-Fligner test for pairwise comparisons [DSCF]: − 12.450, *P* < 0.0001), which, in turn, was substantially lower than that obtained when there were more containers treated with the oil (40.70 ± 4.88 eggs; range: 0–107 eggs; DSCF: 13.184, *P* < 0.0001). Individual females also retained arithmetically fewer eggs in the water-based no-choice trial than in the AMF-based no-choice trial (25.94 ± 3.98 eggs, range: 0–71 eggs), which in turn recorded significantly more eggs retained per female than in the equal-choice trial (DSCF: 9.195, *P* < 0.0001).

**Table 1:** Comparative oviposition responses of *Aedes aegypti* females to Aquatain® Mosquito Formulation-treated water at different levels of competition with water

Oviposition arena^a^	Initial number of females	Number of eggs				Total number of eggs deposited
		Cage 1	Cage 2	Cage 3	Cage 4	
a. [WA1, WA2, W3, W4]	48	509	465	577	533	2084
b. [AQ1, AQ2, WA1, WA2]	48	327	310	304	244	1185
c. [AQ1, AQ2, AQ3, WA1]	48	284	116	287	96	783
d. [AQ1, AQ2, AQ3, AQ4]	48	102	169	138	131	540

^a^a: Water-based no-choice setting; b: equal-choice setting; c: more options in Aquatain® Mosquito Formulation-treated containers; d: Aquatain®-based no-choice trial

**Table 2:** Egg retention responses of *Ae. aegypti* females to Aquatain® Mosquito Formulation-treated water at different levels of competition with water

Oviposition arena^a^	Initial number of females dissected	Number of eggs retained				Total number of eggs retained
		Cage 1	Cage 2	Cage 3	Cage 4	
a. [WA1, WA2, W3, W4]	48	251	171	118	109	649
b. [AQ1, AQ2, WA1, WA2]	28	114	229	119	0	462
c. [AQ1, AQ2, AQ3, WA1]	41	290	490	677	212	1669
d. [AQ1, AQ2, AQ3, AQ4]	38	286	116	185	399	986

^a^See footnote to Table 1

### Oocyte melanization patterns in responses to AMF exposure

**Fig. 3:**
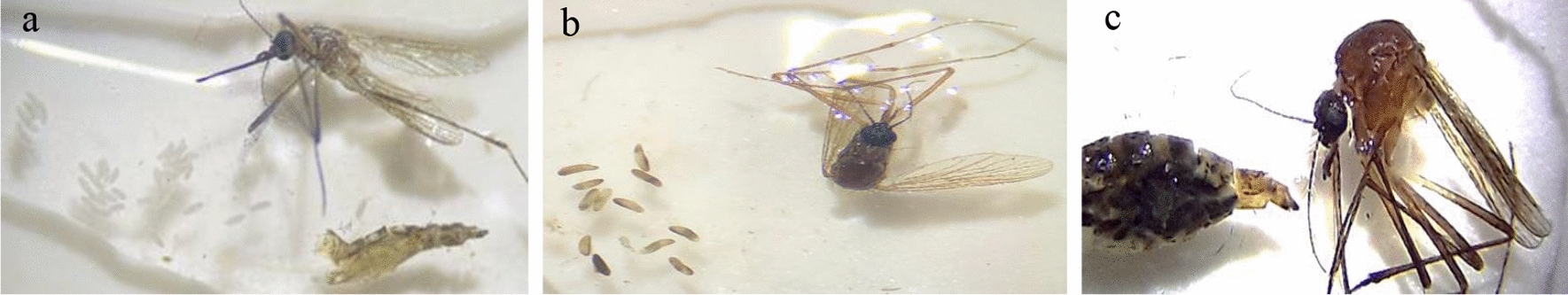
Melanization response patterns of the oocytes of gravid *Ae. aegypti* females after a 10-day exposure to AMF-treated water at various competition levels with water. **a** Non-melanized, **b** partially melanized, **c** fully melanized

Figure 3 shows the egg melanization responses and variations in melanization patterns after a 10-day oviposition period. When water was the only medium present in the oviposition arena and when there was an equal chance for oviposition in two containers with water and two others with AMF-treated water, none of the females subsequently dissected had melanized eggs. Of the 1669 eggs retained by the 41 females provided with additional oviposition opportunities in AMF-treated containers, 76.45% (1276/1669) and 15.27% (255/1669) were partially and fully melanized, respectively. When containers treated with AMF were the only oviposition sites available, 85.13% (824/968) and 14.87% (162/968) of retained eggs were moderately and fully melanized, respectively. The mean number of partially melanized eggs in the no-choice test involving AMF (21.68 ± 3.63 eggs) was lower than that when egg deposition options were biased towards containers supplemented with AMF (31.12 ± 4.86 eggs), but there was no significant discrepancy between the two mean values (DSCF: − 3.321, *P* = 0.087). Female mosquitoes tended to have less fully melanized eggs when all sites were treated with the oil (4.26 ± 1.44 eggs) than their counterparts maintained in the arena with the sole egg-laying option in water (6.22 ± 1.40 eggs), but the difference was not significant (DSCF: − 3.293, *P* = 0.092).

### Mortality responses of* Ae. aegypti* females during oviposition in the presence of AMF

**Fig. 4:**
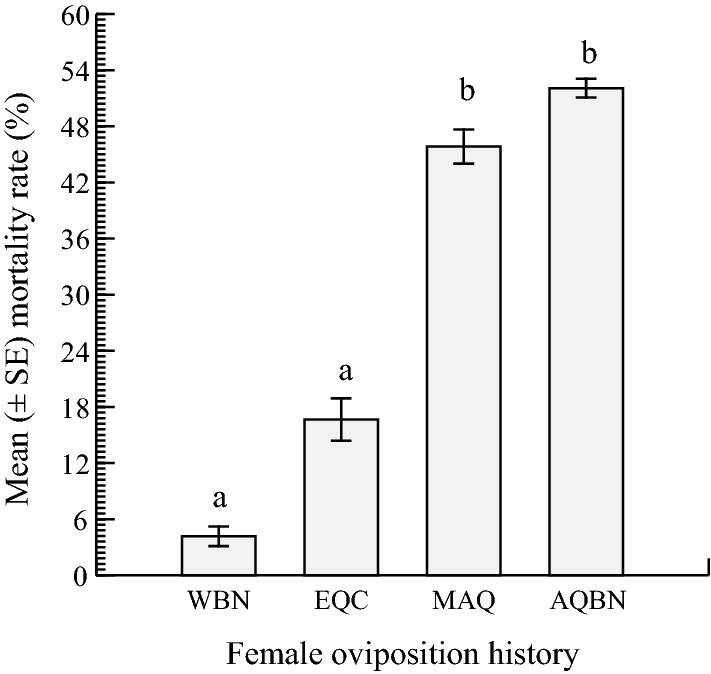
Mortality responses of gravid *Ae. aegypti* females under different oviposition conditions/arena: WBN, EQC, MAQ and AQBN. Bars with the same lowercase letter are not significantly different (*P* < 0.05) based on the Tukey–Kramer HSD test for means comparison.* Abbreviations*: AQBN, AMF-based, no-choice setting; EQC, equal-choice setting; MAQ, more AMF choices setting; WBN, water-based no-choice setting

Significant differences were observed in the survival of *Ae. aegypti* females between the different oviposition opportunities (ANOVA: *F* = 12.690, *df* = 2, *P* < 0.0001; Tukey–Kramer HSD test). When containers with water were the only egg deposition sites available, the mean female mortality was 4.16 ± 4.16% and oscillated between 0 and 16.66%. Arithmetically, this latter mean was far lower than that recorded when females were given equal choices to oviposit in containers with AMF-treated water and two others with water (16.66 ± 4.16%); however, the difference was not statistically significant (Tukey–Kramer HSD test: *P* > 0.05). The mean mortality rate of females in the “more AMF options” experiment (42.83 ± 7.21%, range: 25–58.33%) was significantly greater than that of females in the “equal-choice experiment (Tukey–Kramer HSD test: *P* < 0.05) which, in turn, was numerically lower than that of females in the AMF-based no-choice test. There was no significant difference in female death when there were more options in containers holding AMF-treated water and when only containers with AMF-treated water were present (Tukey–Kramer HSD test, *P* > 0.05) (Fig. 4).

## Discussion

The results of this study revealed significant adverse impacts of the label dose of AMF, the silicone-based monomolecular film, on *Ae. aegypti*. Oviposition responses were reduced with the increased presence of containers with the AMF oil. Egg retention and internal oocyte melanization rates were high in oviposition arenas dominated by the presence of containers with AMF; such effects decreased in the presence of containers with water, especially under the water-based, no-choice conditions. Death occurred at greater rates among females maintained in arenas with an increased presence of oviposition sites treated with AMF compared to arenas with an increased presence of water.

The appeal or lack of appeal (repulsiveness effect) of competitive egg deposition sites to gravid mosquito females is dictated by a diversity of factors, among which the most important is the physico-chemistry of the water medium [54] and the prospect of unfavorable conditions for larval development completion [20, 55]. Various physical elements have been shown to prevent egg deposition in mosquitoes [20, 56, 57], with water surface tension being a particularly critical factor influencing oviposition [22]. Water tension typically occurs when oil is added to water, following which the layer of water molecules and that of oil molecules achieve a balanced force between the two interfaces, causing the reduction of the surface tension at the water surface [35] and the apparition of a film [32]. *Aedes aegypti* has been reported to select oviposition sites that increase the possibility of larval development completion [22–24, 55]. In mosquitoes, the presence of an oil film on the surface of the water of potential breeding habitats has often been associated with reduced survival. For example, Mbare et al. [50], in their study of *Anopheles* vectors, noted a 90% larval mortality and > 80% inhibition of adult emergence at sites with an AMF film. Dawood et al. [32], working with *Culex pipiens*, investigated the effects of AMF on different immature stages. These authors reported significantly greater mortality rates among first-, second- and fourth-instar larvae and pupae in the treated containers than untreated ones, a result they attributed to the ability of AMF to alter the water surface tension. In a recent study, Kavran et al. [49] examined the lethality of AMF on the immature forms of two mosquito species and reported higher mortality rates among juveniles of the dengue vector, *Aedes albopictus.* In an earlier study, Ngrenngarmlert et al. [58] assessed the lethal potential of a silicone-based monomolecular film against *Ae. aegypti* and reported significant larval and pupal mortality rates. In addition to affecting the immature stages, the presence of oil can also affect the behaviors of adults [48], including ovipositing females [43]. The mechanism by which this behavioral change is effected has been well documented. According to Likura et al. [59], mosquito legs are highly hydrophobic, causing a weight-supporting force on water surfaces. For a successful egg-laying to occur, a gravid female must generate a repulsive force from the legs to be able to use the surface of the water as a foothold [58, 59] and evaluate its quality [20]. However, when silicone oil is layered over the water surface, it generates an attractive capillary force that drags the legs towards the water [58], inducing a short contact time and an escape response [59]. A shortened contact time and startle behavior will result in an incomplete exploration of the quality of the oviposition site and thus a decreased probability of egg deposition.

 Two colorless oviposition media were used in the present study: AMF-treated water (150 ml of water + 3.316 µl AMF) and water (150 ml + 3.316 µl). All of the containers used in oviposition bioassays had a similar configuration, and no food was added to any container; as such, differences in oviposition responses due to a difference in study configuration is unlikely. Based on earlier reports and our methodology, the observed low oviposition responses in AMF-treated containers could be explained by an attempt of the gravid females to maximize survival of both offspring and themselves. It is possible that the ovipositing *Ae. aegypti* females associated the presence of AMF with poor nutritional quality and reduced chances of larval development completion. It is also likely that AMF reduced the water surface tension, which in turn prevented gravid females from either landing or staying long enough on the surfaces of the medium in the container to release their eggs.

The egg retention rates in *Ae. aegypti* females placed in environments dominated by the presence of containers treated with AMF were far higher than those of females kept in arenas with only water as oviposition medium. Several studies have reported egg retention in mosquitoes relative to characteristics of oviposition. Chadee et al. [60], working with  *Ae. aegypti*, reported increased egg retention behavior at 7 days post-blood meal when females were not provided with egg-laying substrates.

In their study of a dengue vector, Satho et al. [24] assessed changes in its oviposition responses in no-choice and choice bioassays involving different concentrations of coffee extract. These authors observed that *Ae. albopictus* females withheld increased numbers of mature eggs when the experimental cups containing a highly concentrated extract were the only available sites. Seenivasagan et al. [23] reported that the topical repellent diethyl phenylacetamide deterred egg depositions by *Ae. aegypti* females, which then retained around half of their egg production. Xue and colleagues [61] investigated oviposition behaviors in *Ae. albopictus* in response to DEET (diethyltoluamide) and noted that the females retained high numbers of mature eggs. Bibbs et al. [62] observed that when egg deposition sites were contaminated with a fast-acting pyrethroid insecticide (Transfluthrin), gravid females of dengue vectors withheld almost half of their eggs. Seenivasagan et al. [23] and Bibbs et al. [62] tested *Ae. aegypti* and reported 49% and 50% oviposition deterrence, respectively. In the present study, we looked at the effect of AMF on *Ae. aegypti* females and obtained egg retention rates ranging from 64.61% to 68.06%, which represent deterrence outcomes similar to those in the above-mentioned studies. Egg retention comes with many disadvantageous physiological and behavioral effects [63], including egg resorption [64], initiation of subsequent vitellogenic cycle [65], morphological changes in the follicle [66] and changes in the visual, olfactory and tactile responses of mosquitoes [67] and egg dissemination pattern [60].

Although we did not assess these effects, our study revealed some information on melanization, which is known to play various crucial roles in insects [68]. There was a clear link between oviposition medium type and eggshell melanization level. No oocyte melanization occurred when gravid females were exposed to an equal number of containers of water or AMF-treated water (i.e. equal balance between media). In contrast, eggs dissected from the ovaries of females exposed to an increased number of containers containing AMF-treated water (vs water only) showed a high prevalence of melanized eggs; while the retained eggs of mosquitoes exposed exclusively to AMF-treated water exhibited different levels of melanization (whitish/light gray [non-melanized]; dark-gray with black spots [partially melanized]; and black [fully melanized]). In dengue vectors, this biochemical process [69] generally starts after oviposition [70, 71]. Newly laid eggs that are whitish and smooth [72] undergo a melanization/sclerotization process [73] to become gradually black and hard after 2 to 4 h [72, 74, 75]. During the process, a cascade of enzymatic synthesis and activities involving the phenoloxidase [75] and DOPA decarboxylase [70, 76] lead to the production of melanin. Isoe et al. [71] recently assessed the genetic aspects of melanogenesis in *Ae. aegypti* and found that the eggshell organizing factor 1 (EOF1) plays an essential role in melanization. These authors suggested that partial melanization prior to oviposition is caused by the loss of EOF1 activity, which alters the chemical balance within the oocytes, which in turn activates other eggshell components to initiate the melanization process prematurely. They also noted that EOF1-deficient mosquitoes have eggs with different melanization levels, varying from non-melanized to completely melanized eggshells. In the present study, gravid *Ae. aegypti* females were presented with AMF-treated water as oviposition medium at different competition levels with water for the same length of time (10 days).

Although we did not investigate these phenomena, contingent on the findings of these previous studies, we presume that the synthesis and levels of phenoloxidase and decarboxylase were high in the eggs of females exposed solely to AMF-treated water and when three of the four oviposition containers contained AMF. It is plausible that the observed disparities in melanization levels between eggs in females exposed to AMF-treated water and eggs in non-exposed/less-exposed females occurred due to the high activity levels of these enzymes. This effect of AMF treatment on the activities of phenoloxidase and decarboxylase was possibly less marked in females with no (water treatment) or reduced exposure (equal-choice and three-fourths [3 of 4] of treatments) to AMF. It is also conceivable that the females exposed solely to AMF-treated water had a higher EOF1 expression level and activity. During the 10-day oviposition period, the mortality rates of gravid females were high in arenas with an increased presence of AMF-treated oviposition containers, especially the “AMF-based no-choice treatment”, compared to the “water-based no-choice treatment,” thus indicating the presence of adulticidal factors in the AMF medium. AMF is a polydimethylsiloxane (PDMS, 80%)-based liquid that is not toxic [32], as indicated by its certification for use in drinking water [77, 78]; therefore, death due to toxic chemicals is unexpected. The observed differences in female mortality responses may have been due to at least two factors. First, reducing the water surface tension in the AMF-treated containers, particularly in the AMF-based no-choice and three-fourths treatments, may have produced strong, attractive capillary forces that dragged the legs of females towards the water, consequently increasing the likelihood of sinking and death. In support of this possibility, on the surface of the water, but not on the surface of water with a silicone oil film layer, female mosquitoes can use the surface as a foothold to lay their eggs, using a maximal repulsive force from their legs [59]. Second, the untimely internal oocyte melanogenesis during egg retention may have induced the production and accumulation of toxic compounds among females with increased exposure to AMF-treated water, which in turn raised the possibility of intoxication. In favor of this contention, it has been shown that phenoloxidase activity in the insect hemolymph can produce quinonoid toxic compounds [79] and that melanin and its quinone intermediates are toxic to hosts [70].

## Conclusions

We observed that treating all, three of four or half (2 of 4) of potential breeding sites of *Ae. aegypti* in a given environment with AMF altered the potential contribution of the site to the next generation. AMF treatment prevented females from laying sizeable numbers of eggs and caused increased egg retention and internal egg melanogenesis, all of which are conducive to the lack of reproductive success. The application of AMF in potential developmental sites also triggered the death of gravid females, thus denoting the adulticidal potential of this film. It is clear that AMF performed as an oviposition deterrent and as an indirect killer of ovipositing *A. aegypti* females; this latter observation would be a supplemental benefit to vector control programs. These attributes, coupled with its previously recognized direct lethality on the immature stages of dengue vectors [49, 58], advocate for the incorporation of AMF into integrated approaches for dengue vector control. As AMF is safe for humans [50, 77, 78], its use in domestic areas where *Ae. aegypti* thrives naturally may be widely accepted. In addition, as the mode of action of AMF is physical, its use and rotation with other larvicides can help mitigate insecticide resistance issues.

## Data Availability

The data supporting the results of this paper are available upon request.
